# CIMAvax-EGF: A New Therapeutic Vaccine for Advanced Non-Small Cell Lung Cancer Patients

**DOI:** 10.3389/fimmu.2017.00269

**Published:** 2017-03-13

**Authors:** Danay Saavedra, Tania Crombet

**Affiliations:** ^1^Center of Molecular Immunology, Havana, Cuba

**Keywords:** non-small cell lung cancer, cancer vaccine, clinical trial, CIMAvax-EGF, immunotherapy

## Abstract

Lung cancer is the common fatal illness with the highest incidence and mortality globally. Epidermal growth factor receptor overexpression by tumor cells is associated with uncontrolled proliferation, angiogenesis, anti-apoptotic signals, metastization, and invasiveness. CIMAvax-EGF vaccine consists of a chemical conjugate of the EGF with the P64 protein derived from the Meningitis B bacteria and Montanide ISA 51, as adjuvant. The vaccine is projected to induce antibodies against EGF that results in EGF withdrawal. CIMAvax-EGF demonstrated to be safe and immunogenic in advanced non-small cell lung cancer (NSCLC) patients. The efficacy study was an open-label, multicentric Phase III clinical trial, which enrolled 405 advanced NSCLC patients. Patients with proven stage IIIB/IV NSCLC, who had completed four to six cycles of chemotherapy (CTP) were randomized to receive CIMAvax-EGF or best supportive care. CIMAvax-EGF resulted in a significantly larger overall survival in patients receiving at least four doses. High EGF concentration at baseline was a good predictive biomarker of the vaccine activity and a poor prognostic biomarker for the non-treated population. The proportion of CD8+CD28− cells, CD4 cells, and the CD4/CD8 ratio after first-line CTP was also associated with CIMAvax-EGF clinical benefit. After completing the Phase III, a Phase IV trial was done where the vaccine was administered in primary care units. Administering the vaccine at primary care institutions granted better access and treatment compliance. Safety was confirmed. Several clinical trials are currently ongoing to validate EGF as a predictive biomarker of CIMAvax-EGF efficacy.

## The Role of Checkpoint Inhibitors (CPIs) in the Control of Non-Small Cell Lung Cancer (NSCLC)

The strategy of triggering the immune system to control tumor progression is not new in cancer research but has been characterized by alternating trends of excitement or frustration. BCG, interferon, and interleukin-2 provided clinical evidences of antitumor activity, but their role in the oncology practice remained limited to few tumor localizations (1, 2). With the advent of immune “check-points” inhibitors, cancer immunotherapy has proven to radically increase the survival of patients bearing advanced melanoma, lymphoma, renal, lung, urothelial, and head and neck tumors (3, 4). Immunotherapy represents an “unconventional” way of treating cancer by targeting the immune system, not the tumor itself (5). The hypothesis is that hindering the “switch-off” receptors like CTLA-4 and PD1 in the lymphocytes, would set the immune system free to destroy cancer. Antibodies against CTLA-4, progressive disease (PD)-1 (programmed death), and PD-1 ligands (PD1-L) represent a major step forward and are the first examples of broadly effective and durable cancer immunotherapies (5, 6).

Lung cancer is the common fatal illness with the highest incidence and mortality globally. NSCLC is the most common histological type of lung cancer (7). Albeit NSCLC is not a classical “immune-sensitive” cancer like melanoma or renal cell carcinoma, two anti-PD1 antibodies and one anti-PD1L antibody have been approved for the treatment for patients with advanced disease.

Nivolumab, a PD-1 CPI, was evaluated in a Phase III study in patients with non-squamous NSCLC that progressed during or after platinum-based doublet chemotherapy (CTP). Overall survival was longer with nivolumab than with docetaxel, a taxane derivative that inhibits the polymerization of microtubules. The median overall survival was 12.2 months in the nivolumab group and 9.4 months in the docetaxel group (8). As well, patients with advanced squamous cell NSCLC who have PD after first-line CTP were randomized to receive nivolumab or docetaxel. The median overall survival was 9.2 months with nivolumab vs. 6.0 months with docetaxel (9).

On the other hand, patients with previously treated NSCLC and PD-L1 expression on at least 1% of tumor cells were randomized to receive pembrolizumab (a different anti-PD1 antibody) at two-dose levels. Overall survival was significantly larger for pembrolizumab, at the two evaluated doses. Median overall survival was 10.4 months with pembrolizumab at 2 mg/kg, 12.7 months after pembrolizumab at 10 mg/kg and 8.5 months after receiving docetaxel. In patients with at least 50% of cells expressing PD-L1, median survival time (MST) was better with pembrolizumab (10).

Moreover, in patients with newly diagnosed stage IIIB/IV NSCLC and PD-L1 expression on 50% of cancer cells, pembrolizumab was associated with significantly longer progression-free and overall survival as compared to platinum-based CTP. A total of 305 patients were randomly allocated to platinum CTP or pembrolizumab. Patients in the pembrolizumab group had a median PFS of 10.3 months, compared to 6.0 months for the CTP group. The 6 months overall survival was 80.2% in the pembrolizumab arm vs. 72.4% in the CTP arm (11).

Finally, FDA has lately accepted atezolizumab (an anti-PD1L antibody) for treating CTP-refractory, metastatic NSCLC patients. The approval followed the findings from the randomized Phase III OAK and Phase II POPLAR clinical trials, indicating a median 4.2 months survival advantage over docetaxel CTP (MST in OAK trial: 13.8 vs. 9.6 months). OAK study participants included patients with varying PD-L1 status and both squamous and non-squamous tumors (12, 13).

In summary, three immunomodulatory drugs, two anti-PD1 antibodies (nivolumab and pembrolizumab), and one anti-PD1 ligand antibody (atezolizumab), have shown to improve the survival of advanced NSCLC, still considered an unmet medical need. Table 1 summarizes the most important results of the three CPIs approved so far for second- or first-line therapy of advanced NSCLC patients.

**Table 1:** **CPIs in the treatment of patients with advanced NSCLC**.

Patient population	CPI arm	Control arm	MST	
			CPI arm (months)	Control arm (months)
Non-squamous NSCLC patients that progressed during or after platinum-based doublet chemotherapy (CTP)	Nivolumab	Docetaxel	12.2	9.4
Squamous NSCLC patients that progressed during or after platinum-based doublet CTP	Nivolumab	Docetaxel	9.2	6
Previously treated NSCLC with progressive disease (PD)-L1 expression on at least 1% of tumor cells	Pembrolizumab (2 mg/kg)	Docetaxel	10.4	8.5
Previously treated NSCLC with PD-L1 expression on at least 1% of tumor cells	Pembrolizumab (10 mg/kg)	Docetaxel	12.7	8.5
CTP-refractory, metastatic NSCLC	Atezolizumab	Docetaxel	13.8	9.6
Previously untreated advanced NSCLC with PD-L1 expression on at least 50% of tumor cells	Pembrolizumab (200 mg)	Carboplatin plus pemetrexed, cisplatin plus pemetrexed, carboplatin plus gemcitabine, cisplatin plus gemcitabine, carboplatin plus paclitaxel	6 months	6 months
			SV rate: 80.2%	SV rate: 72.4%

*NSCLC, non-small cell lung cancer; CPI, checkpoint inhibitor; MST, median survival time*.

## EGF/Epidermal Growth Factor Receptor (EGFR) System and CIMAvax-EGF Mechanism of Action

Oncogenic mutations have arisen as key therapeutic targets for molecular treatments in several cancers (14). EGFR, a well-validated oncogene, is a 170-kDa membrane glycoprotein. The intracellular domain is associated with protein tyrosine kinase activity, and its overexpression by tumor cells alters the regulation of the cell cycle, blocks apoptosis, promotes angiogenesis, and increases the motility and invasiveness of the tumor cells (15).

Therefore, EGFR as well as its downstream mediators have been identified as important therapeutic targets. The approved small-tyrosine kinase inhibitors (TKIs) of EGFR, gefitinib (Iressa™), erlotinib (Tarceva™), and afatinib (tykerb™), are effective in a group of NSCLC patients whose tumors carry stimulating mutations within the kinase domain of EGFR (16–19). EGFR–TKIs are the best option as front-line therapy in EGFR mutant NSCLC patients. In pretreated NSCLC, EGFR–TKIs are more effective than conventional cytotoxic therapy, in existence of EGFR mutations (16–19). EGFR has seven known ligands, among which, EGF is one of the most critical (20, 21).

The strategy of “sequestering” EGF reproduces the “hormonal castration” therapy, known to be effective in hormone-dependent tumors such as breast and prostate, thus extending this concept to other types of malignant tumors.

**Figure 1:**
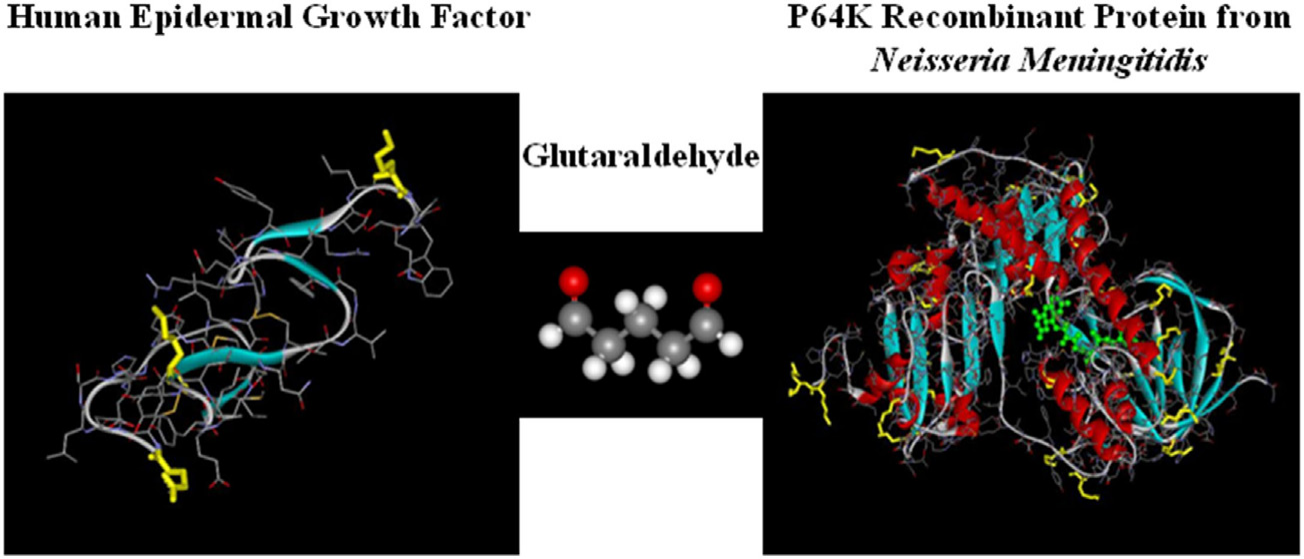
**CIMAvax-EGF composition**. CIMAvax-EGF therapeutic vaccine consist on a chemical conjugate of the EGF with the P64K protein derived from *Neisseria meningitidis*.

The mechanism of action of CIMAvax-EGF consists on the formation of antibodies against EGF, breaking the tolerance to a self-protein. This is possible because the vaccine consists on a chemical conjugate of the recombinant EGF with the P64k protein derived from the *Neisseria meningitidis* (conjugate EGF-P64K) (Figure 1) and the adjuvant Montanide ISA 51 (22). CIMAvax-EGF is administered by the intramuscular route, at four injection sites (22, 23).

CIMAvax-EGF vaccine exerts its anti-cancer activity by targeting the immune system, inducing anti-EGF antibodies that result in the decline of the circulating EGF in sera (23, 24). This, in turn, significantly decreases the probability that the remaining EGF binds to its receptor (EGFR) on the surface of cancer cells. EGF withdrawal results in the loss of a key pro-proliferation and pro-survival signal for the neoplastic cells (23, 24). The vaccine has demonstrated to be safe and immunogenic in more than 5,000 advanced NSCLC patients (23, 24).

CIMAvax-EGF was approved as a maintenance treatment for patients with stage IIIB/IV NSCLC, after front-line CTP.

Two randomized studies have been completed so far. The Phase II clinical trial included 80 advanced NSCLC patients: 40 vaccinated and 40 treated with supportive care. Patients joined the trial after finalizing first-line CTP, regardless their objective response. CIMAvax-EGF was non-toxic and induced anti-EGF antibodies. Vaccinated subjects showed a trend toward better survival, which was not statistically significant at this sample size (25).

The efficacy study consisted in an open-label, multicentric Phase III clinical trial, which enrolled 405 advanced NSCLC patients, at 21 research sites. Patients with proven stage IIIB/IV NSCLC, who received four to six cycles of platinum-based CTP were randomized to vaccine arm [CIMAvax-EGF plus best supportive care (BSC)] or to control arm (BSC alone). Primary endpoint was overall survival while secondary endpoints were the assessment of serum EGF concentration, immunogenicity, and safety. All lung cancer patients completed front-line CTP achieving stable disease, partial, or complete response of the target lesions. Most subjects had cisplatin/carboplatin in combination with vinblastine, etoposide, or paclitaxel. Randomization (EGF cancer vaccine vs. BSC) was unbalanced (2:1), given the preliminary evidence of survival advantage shown in the Phase II study. Vaccine schedule consisted in four biweekly doses (induction phase) followed by monthly reimmunizations (maintenance). Cyclophosphamide was administered before vaccination at a low, immunomodulatory dose (200 mg/m^2^). Vaccination was maintained until severe patient condition worsening (PS = 3) or unmanageable toxicity (26).

This study was registered in the National Public Registry of Clinical Trials; a WHO-validated public registry (http://www.who.int/ictrp/network/rpcec/en, RPCEC00000161). In total, 270 vaccinated and 135 controls were enrolled in the Phase III study. Both groups were well balanced according to the most important prognostic variables. The majority of the patients were men, current, or past smokers, with an ECOG performance status of 1. The most prevalent histology was squamous cell carcinoma, and they had stable disease or partial response after first-line platinum doublet. Vaccination was safe, and the most common adverse reactions were mild or moderate injection site events, fever, headache, chills, vomiting, and general malaise. CIMAvax-EGF significantly augmented overall survival when the Harrington–Fleming test was applied (26). The Harrington–Fleming is a weighted log-rank test that can be used once the non-proportionality of the hazard ratio is confirmed (27, 28). This waited log-rank is the ideal test when there is a deferred split of the time to event curve (27, 28). This is the case of therapeutic cancer vaccines or immune-modulatory drugs, which effect may manifest several months after the intervention. In this scenario, the projected hazard ratio does not apply from the beginning but at the separation of both curves. MST was 10.83 months for vaccinated vs. 8.86 months for non-vaccinated. In the Phase III trial, the 5-year survival rate was 14.4% for vaccinated subjects vs. 7.9% for controls. The advantage was larger in those patients that completed vaccination induction consisting in four doses (“per protocol” scenario). The “per protocol” scenario is very relevant for CIMAvax-EGF given that several doses are required to break the tolerance and induce a protective response. MST was 12.43 months for vaccinated subjects completing induction vs. 9.43 months, for control patients (Table 2). Those controls that did not survived for at least 42 days (vaccine induction time) were excluded from the analysis. The 5-year survival rate was 16.62% for vaccinated patients vs. 6.2% for controls. A subgroup analysis considering demographic or tumor variables was done, and the larger gain was seen in smoker patients bearing squamous cell carcinomas with an ECOG 1 (26).

**Table 2:** **CIMAvax-EGF in the treatment of patients with advanced NSCLC (Phase III clinical trial)**.

Patient population	CIMAvax-EGF arm	Control arm	MST	
			CIMAvax arm (months)	Control arm (months)
Stage IIIB/IV NSCLC patients, with at least stable disease after CTP (ITT)	CIMAvax-EGF	BSC	10.83	8.86
Stage IIIB/IV NSCLC patients, with at least stable disease after CTP (PP)	CIMAvax-EGF	BSC	12.43	9.43
Stage IIIB/IV NSCLC patients, with at least stable disease after CTP. Patients with (EGF) > 870 pg/ml	CIMAvax-EGF	BSC	14.66	8.63

*NSCLC, non-small cell lung cancer; MST, median survival time; PD, progressive disease; CTP, chemotherapy; BSC, best supportive care*.

## CIMAvax-EGF Immunogenity and Predictive Biomarkers of Efficacy

**Figure 2:**
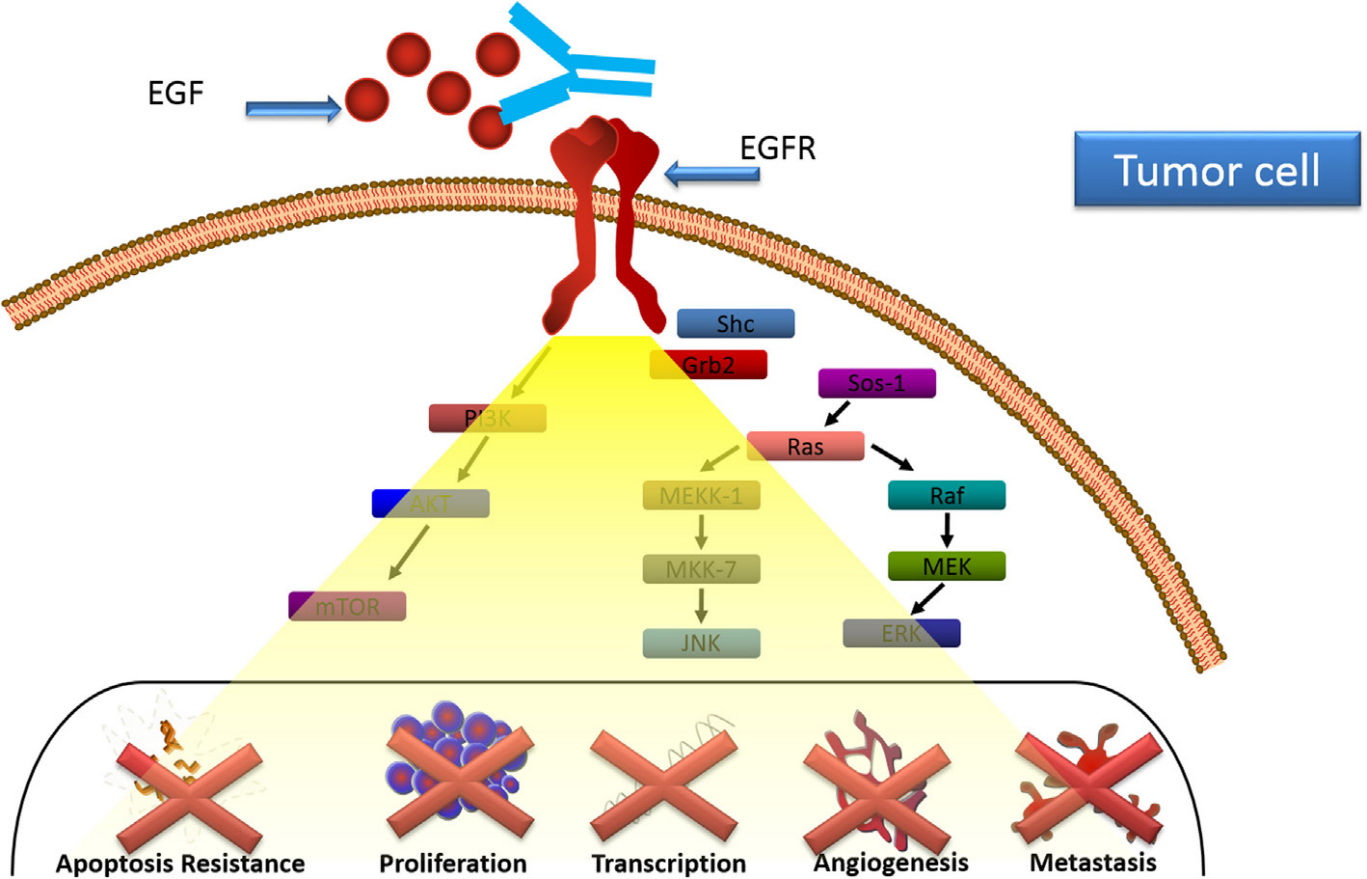
**CIMAvax-EGF mechanism of action**. Anti-EGF antibodies induced by CIMAvax-EGF inhibit EGF–epidermal growth factor receptor (EGFR) binding and abrogate EGFR activation.

Immune response was characterized in patients treated with CIMAvax-EGF (24). Anti-EGF antibodies induced by CIMAvax-EGF inhibited EGF–EGFR binding and abrogated EGFR activation (Figure 2). After immunization, there was a decrease in the circulating EGF which was inversely correlated with the antibody response. Antibody response also correlated with survival benefit since those patients displaying higher antibody titers exhibited better survival (24).

In the Phase III trial, a large proportion of patients (78.8%) met the good antibody response (GAR) condition (anti-EGF antibody titers ≥ 1:4,000 sera dilution). GAR condition was associated with longer survival in the preceding exploratory and Phase II trials. The geometric mean of the maximum antibody titers was 1:12,646 sera dilution, while the maximum anti-EGF titer was 1:1,024,000. Patients developing a GAR as soon as day 32 had a significant survival benefit (MST = 27.28 months) as compared to controls (26).

The functionality of anti-EGF antibodies was also evaluated. Sera from vaccinated patients inhibited the binding between EGF and its receptor. Median binding inhibition capacity was 20 and 40% after 5 and 12 months from vaccination, respectively (24). Furthermore, post-immune sera abrogated EGFR phosphorylation. Median phosphorylation inhibition was 65 and 85% after 5 and 12 months, respectively (24).

To discern the immune dominance of the antibody response induced by vaccination, several peptides mimicking the main EGF epitopes were synthesized. Sera from vaccinated patients were then tested for binding to the peptides in an enzyme-linked immunosorbed assay. In the Phase III study, 46% of the patients showed an immune-dominant response against the loop B of the EGF molecule (26).

The immune response of 19 long-term (more than 2 years) NSCLC survivors, regularly treated with CIMAvax-EG, was assessed (29). Previous studies showed that the anti-EGF antibody titers increased in vaccinated patients after repeated immunizations, until a plateau is reached (24–26). In long-term vaccinated patients, the anti-EGF antibody response remained high, reaching a plateau at 1:10,000 sera dilution. Although a deferred decrease in antibody titers was found in one third of the uninterrupted vaccinated patients, for the majority (two-thirds), there was no evidence of clonal exhaustion after 2 years of monthly vaccination. The immunodominance of the antibody response induced by CIMAvax-EGF was tested in long-term vaccinated subjects. The predominant response was against the loop B, which is the main binding site of EGF to EGFR. Long-lasting vaccination resulted in a reduction of serum EGF level. EGF concentration decreased to undetectable values in all continued vaccinated patients (29).

In summary, prolonged vaccination with CIMAvax-EGF induced high anti-EGF antibodies, capable to maintain serum EGF in undetectable levels. Toxicity was not exacerbated with lengthy vaccination. Long-term “EGF deficiency” did not result in deleterious effect for normal tissues. Previously, it was published that the lack of EGF produces delayed development of fetal tissue but no injury on healthy adult tissues (30).

During the last decade, the scientific community has been working hard on the development and evaluation of biomarkers for cancer drug development (31).

Several attempts have been done to find predictive biomarkers of clinical benefit of CIMAvax-EGF. Vaccinated patients with serum EGF concentration >870 pg/ml showed larger survival as compared with controls with the same EGF serum level. MST in this patient population was 14.66 months, as large as the survival of patients receiving other drugs as continuation or switch maintenance (26). MST was 8.63 months for those control patients with EGF concentration greater than 870 pg/ml (Table 2). Five-year survival rate for patients with high (EGF) was 23% for vaccinated patients, while no controls were alive at the referred time interval. The association between EGF levels and prognosis remained significant when the prognostic variables (gender, smoking history, performance status, and staging) were included in the multivariate analysis (26).

On the other hand, control patients with a high (EGF) had a significantly shorter survival (8.63 months) as compared with non-treated subjects with low (EGF) at baseline (15.06 months). In summary, the Phase III trial demonstrated that the EGF level in patients’ sera could be simultaneously a biomarker of poor prognosis and a predictive factor of CIMAvax-EGF benefit. This result confirms the role of the EGF in the biology of the tumor but also provides a biomarker for selecting patients who benefit largely from vaccination with CIMAvax-EGF (26).

The impairment of immune system of cancer patients induced by the tumor together with the previous oncological therapies is largely proven. The evaluation of immunocompetence would provide evidences of which patients are going to benefit from immunotherapy (32). A deficit in the number of B cells, a reduced CD4/CD8 ratio and an increase in late-stage differentiated cells such as CD8+CD28− T cells distinguish the “immune-compromised” profile (33). In that logic, besides EGF concentration, the proportion of CD8+CD28− T cells, CD4 T cells, and the CD4/CD8 ratio after CTP was correlated with the clinical benefit of CIMAvax-EGF (33).

Vaccinated patients with CD4+ T cells counts greater than 40%, CD8+CD28− T cells counts lower than 24% and a CD4/CD8 ratio >2 after first-line platinum-based CTP, achieved a significantly large median survival, as compared to controls with the same phenotype. MST was 46.4 months for vaccinated patients with CD4+ counts >40% vs. 12.3 months for the matched controls, 37.2 vs. 14.3 months for vaccinated and controls with CD8+CD28− T cells counts <24% and 50.4 vs. 14.3 months for treated vs. non-treated patients with CD4/CD8 ratio >2 (33).

These findings highlight the potential value of T cell subpopulations and EGF serum levels, measured after front-line CTP, as predictive biomarkers of CIMAvax-EGF efficacy.

## Combining CTP and CIMAvax-EGF

CIMAvax-EGF is commonly administered after patients have finished first-line CTP. However, it would be important to start vaccination earlier in the course of the disease, given that the vaccine requires time to elicit a neutralizing response. In that sense, CIMAvax-EGF was administered concurrently with platinum doublets or even, before CTP (34). In addition, CTP and cancer vaccines could be additive through different mechanisms: by decreasing immunosuppressive cells such as T-regulatory and myeloid-derived suppressor cells, by stimulating massive antigen release leading to effective cross-priming, by modifying the tumor microenvironment and by augmenting the T-cells traffic of into the tumors (35). Oxaliplatin and cisplatin can stimulate antitumor responses, through the induction of immunogenic cell death (35). The release of new antigens can activate dendritic cells, which in turn, that activate cytotoxic lymphocytes (35, 36).

Dose-dense platinum CTP did not affect CIMAvax-EGF capacity to induce a potent antibody response. Immunogenicity in terms of percentage of good responders or immunodominance against loop B was better after vaccinating concurrently or before CTP, as compared to the standard sequential platinum doublets and vaccination (34). Increased immunogenicity could be explained by the earlier unset of vaccination or by the potentiating effect of the cytotoxic drugs.

## CIMAvax-EGF in Primary Care Units and Future Perspectives

After completing the Phase III, a Phase IV trial was launched where the family medicine physicians administered CIMAvax-EGF in primary health care units (policlinics). In total, 45 primary level units together with 24 secondary level units (hospitals) participated in the study that enrolled more than 1,000 patients in 3 years. This study was registered in the National Public Registry of Clinical Trials (http://www.who.int/ictrp/network/rpcec/en, trial number RPCEC00000181). Administering the vaccine at primary care institutions granted better access and treatment compliance. Safety was confirmed; the most frequently reported adverse events were pain at the site of injection followed by fever, headache, chills, nausea, and dyspnea (22).

Overall survival of those patients that received at least one vaccine dose was 13.9 months (mean) and 7.0 months (median). Survival rate at 12 and 24 months was 34.8 of 18.1%, respectively. On the other hand, the overall survival of patients receiving at least the induction doses was 16.93 months (mean) and 9.9 months (median). The 12 and 24 months survival rate was of 44.1 and 23.3%, respectively.

In summary, CIMAvax-EGF was safe in patients with NSCLC at advanced stages treated in primary care facilities. The safety profile coincided with the previously described in controlled studies. CIMAvax-EGF also showed benefit in terms of survival, mainly in those subjects that completed four vaccine doses. Treatment with CIMAvax-EGF resulted in preliminary evidences of improvement in the quality of life, which was significant for the emotional functioning and the fatigue symptom. The use of medications to control pain was stable during vaccination (22).

Several clinical trials are currently ongoing. A new Phase III trial (WHO-validated public registry; http://www.who.int/ictrp/network/rpcec/en, trial number RPCEC00000208) is open for enrollment, where CIMAvax-EGF is used as switch maintenance in patients completing front-line CTP that has EGF concentration higher than 870 pg/ml (enrichment design). The main goal of the trial is to prospectively validate EGF as a predictive biomarker. In this scenario, the randomization is unbalanced (3:1) given the previous evidences of the clinical benefit of the vaccine. In addition, a new Phase IV (WHO-validated public registry; http://www.who.int/ictrp/network/rpcec/en, trial number PCEC00000205) was launched in 178 policlinics (at least one investigation site per state municipality) and 25 hospitals. Patients will be recruited by the oncologists in the specialized oncology services, but will be treated in their neighborhood, at the primary health care facilities. The aim is to grant vaccine access and to improve treatment compliance. In this trial, EGF concentration will be measured but not as an inclusion criterion. Instead, EGF at baseline will be retrospectively correlated with the clinical efficacy. An EGF quantification system was developed in the country by the National Center for Immunoassay, to accompany the vaccine prescription (37). Both studies will permit the consolidation of the scientific evidence of the EGF as a biomarker. Other translational studies are planned to gather more information on the relevance of the lymphocyte subpopulation as well as the individual tumor biology (mainly associated with EGFR mutations) for the CIMAvax-EGF efficacy.

## Author Contributions

DS was involved in the evaluation of immunogenicity and predictive biomarkers of CIMAvax-EGF efficacy (EGF concentration and immunophenotyping). TC was involved in trials’ design and implementation. Both authors participated in the analysis, writing, and revision of the manuscript.

## Conflict of Interest Statement

Both authors, DS and TC, are employees of the Center of Molecular Immunology, the institution that owns the patent and manufactures CIMAvax-EGF. Neither author receive additional compensation associated with CIMAvax-EGF registration or marketing.
